# Prediction of Mature MicroRNA and Piwi-Interacting RNA without a Genome Reference or Precursors

**DOI:** 10.3390/ijms16011466

**Published:** 2015-01-08

**Authors:** Mark S. Menor, Kyungim Baek, Guylaine Poisson

**Affiliations:** Department of Information and Computer Sciences, University of Hawaii at Mānoa, 1680 East-West Road, Honolulu, HI 96822, USA; E-Mail: mmenor@hawaii.edu

**Keywords:** microRNA, piwi-interacting RNA, non-coding RNAs, kernel machine, classification

## Abstract

The discovery of novel microRNA (miRNA) and piwi-interacting RNA (piRNA) is an important task for the understanding of many biological processes. Most of the available miRNA and piRNA identification methods are dependent on the availability of the organism’s genome sequence and the quality of its annotation. Therefore, an efficient prediction method based solely on the short RNA reads and requiring no genomic information is highly desirable. In this study, we propose an approach that relies primarily on the nucleotide composition of the read and does not require reference genomes of related species for prediction. Using an empirical Bayesian kernel method and the error correcting output codes framework, compact models suitable for large-scale analyses are built on databases of known mature miRNAs and piRNAs. We found that the usage of an *L*_1_-based Gaussian kernel can double the true positive rate compared to the standard *L*_2_-based Gaussian kernel. Our approach can increase the true positive rate by at most 60% compared to the existing piRNA predictor based on the analysis of a hold-out test set. Using experimental data, we also show that our approach can detect about an order of magnitude or more known miRNAs than the mature miRNA predictor, miRPlex.

## 1. Introduction

Non-coding RNAs (ncRNAs) are functional RNA transcripts that do not translate into proteins. The small ncRNA class consists of ncRNAs of lengths of about 20 to 30 nucleotides (nt). Two types of small ncRNA of particular interest are microRNA (miRNA) and piwi-interacting RNA (piRNA), as they play important roles in post-transcriptional regulation and are implicated in many essential biological processes. The discovery of novel miRNA and piRNA is therefore an important step in the understanding of these processes.

The mature form of miRNA regulates messenger RNAs (mRNAs) in plants and animals as a form of the post-transcriptional regulation of genes. This is accomplished by the binding of the mature miRNA to the target mRNA transcript, typically within the 3' untranslated region (UTR) [1]. Therefore, miRNAs play key roles in the regulation of cellular processes, and their dysregulation is implicated in many diseases, such as cancer [2].

Mature miRNA arises from a complex biogenesis. The canonical pathway to a mature miRNA involves the transcription of a long primary RNA transcript (pri-miRNA). The Drosha enzyme cleaves the pri-miRNA, resulting in a shorter hairpin structure, called the precursor miRNA (pre-miRNA). Alternative non-canonical biogenesis pathways are known to produce pre-miRNA without Drosha. For example, mirtrons are miRNAs formed by the introns of a host protein coding gene [3]. In any case, the pre-miRNA is further cleaved into two segments by the enzyme, Dicer. The now liberated double-stranded stem of the pre-miRNA has historically been called the miRNA/miRNA* duplex. The duplex is later separated, and both strands may possibly lead to functional, mature miRNA by recruitment into a RNA-induced silencing complex (RISC) via Argonaute proteins.

On the other hand, the most highly expressed and most diverse small ncRNAs in animals are the piRNAs [4,5]. Mature piRNAs form RNA-protein complexes with piwi proteins. The piRNA complexes are linked to epigenetic and post-transcriptional silencing of retrotransposons, particularly in the germ line cells, and with tumorigenesis [6]. However, recent evidence suggests further roles for piRNA beyond transposon silencing, such as piRNAs that target genes involved with establishing memory in neurons [7]. Many details of piRNA, such as the target silencing mechanisms and aspects of its biogenesis, remain open questions [8]. Therefore, piRNAs are important, and continuing research will further establish their role in cellular processes.

High-throughput small RNA sequencing technologies have been developed and helped in the study of small ncRNAs. For example, small RNA sequencing technologies were used to implicate piRNAs in anti-viral defense in mosquitoes [9]. A variety of methods have been developed to annotate the small RNA reads that require genomic information, such as miRCat [10] and miRDeep [11,12], for miRNA identification and prediction. Nevertheless, annotation of small ncRNA datasets remains a challenge, as for example studies on locust small RNA datasets leave 30%–40% of the sequences unannotated. Furthermore, the requirement of genomic information prevents the majority of methods from being used on non-model organisms, where whole-genome sequencing and annotation is a non-trivial hurdle.

For miRNA, there are two existing methods for predicting mature miRNA from small RNA sequencing without a genome reference: MiRMiner [13] and miRPlex [14]. MiRMiner relies on evolutionary conservation and uses the genomic information from other species as a substitute for direct genome reference. Thus, MiRMiner requires genome references reasonably close to the target species. On the other hand, miRPlex exploits the knowledge of miRNA biogenesis by predicting possible miRNA/miRNA* duplexes via RNA folding algorithms. This avoids the use of any genome references. However, the use of miRPlex on the full small RNA datasets that have millions of reads would require a powerful parallel computing infrastructure, due to the very large number of possible pairs that need to be evaluated. To reduce the number of duplexes, miRPlex primarily filters the sequences considered by abundance. This can lead to the omission of miRNAs that were not highly expressed.

For piRNA, there exists a method, called piRNApredictor, for predicting mature piRNA from small RNA sequencing data [15]. piRNApredictor is based purely on nucleotide composition and does not require reference genomes nor sequencing of precursor forms. However, our previous study suggests that the predictive performance of piRNApredictor can be significantly improved [16].

In this work, we propose an approach that addresses the weaknesses of existing methods and improves our previous approach presented in [16]. For input data representation, new sequence features are included in addition to the *k*-mer features used in the previous work, and a feature selection method is employed for dimensionality reduction and performance improvement. We also consider an additional Gaussian kernel utilizing the *L*_1_ distance metric in the proposed approach. For experimental evaluation of the proposed method, we updated the miRNA dataset using the latest release of miRBase and formed a new dataset for other small ncRNAs (non-miRNA and non-piRNA) using a small RNA dataset from the NCBI Genetic Expression Omnibus (GEO) database, which would provide a more realistic representation of the data being present in the small RNA sequence project. The piRNA dataset remains the same as used in the previous work, since the update in the latest version of the NONCODE, a noncoding RNA database, expanded the long ncRNA collection, which would be irrelevant to our work. The method proposed in this study will rely primarily on the nucleotide composition of the read, thereby avoiding the need of reference genomes and the computationally expensive pairwise folding of the reads required by existing methods. We use multiclass relevance units machine (McRUM) [16], an empirical Bayesian kernel method for classification, to achieve compact models appropriate for large-scale analyses. The results suggest a significant improvement over our previous efforts and to existing methods.

## 2. Results and Discussion

### 2.1. Feature Selection

Since mature miRNAs and piRNAs lack strong secondary structures, we analyze the nucleotide composition of the ncRNAs. Our previous analysis [16] suggests that this composition approach suffices for identifying piRNAs, but is not optimal in the identification of miRNAs. To improve the performance with regards to miRNAs, we consider additional miRNA-centric features, such as the first nucleotide of the sequence, the composition of the seed region and the A/U composition. We use correlation-based feature selection (CFS) [17] to select a subset of features on which to build classifier models. Details on features are described in Section 3.4.

In total, we consider 1389 features, including the 1364 unique *k*-mers, for *k* = 1 to 5, that represent nucleotide composition. For the CFS McRUMs, CFS is used to select a subset of features to build a McRUM for each fold of cross-validation. CFS selects about 142–161 features per fold, which is about a ten-fold decrease from using all features. When applied to the entire training dataset, CFS selects 154 features, indicating that the majority of the features are redundant. In all cases, among those selected by CFS are the four binary features representing A, C, G and U, which mark the identity of the first nucleotide. This is not surprising, as both miRNA and piRNA are biased toward starting with a U base. Also selected is the AU score [18], as both piRNA and miRNA tend to have higher scores. This too is not surprising for miRNA, because miRNA targets are known to have a high AU content [18]. Lastly, the frequency of the two-mer CG in the potential seed region is selected. Interestingly, this feature distinguishes piRNA rather than miRNA, as piRNAs are more biased toward lower scores than other ncRNAs.

**Figure 1:**
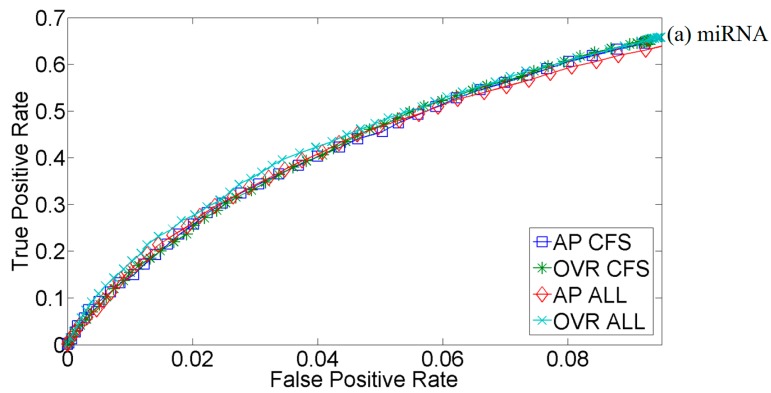

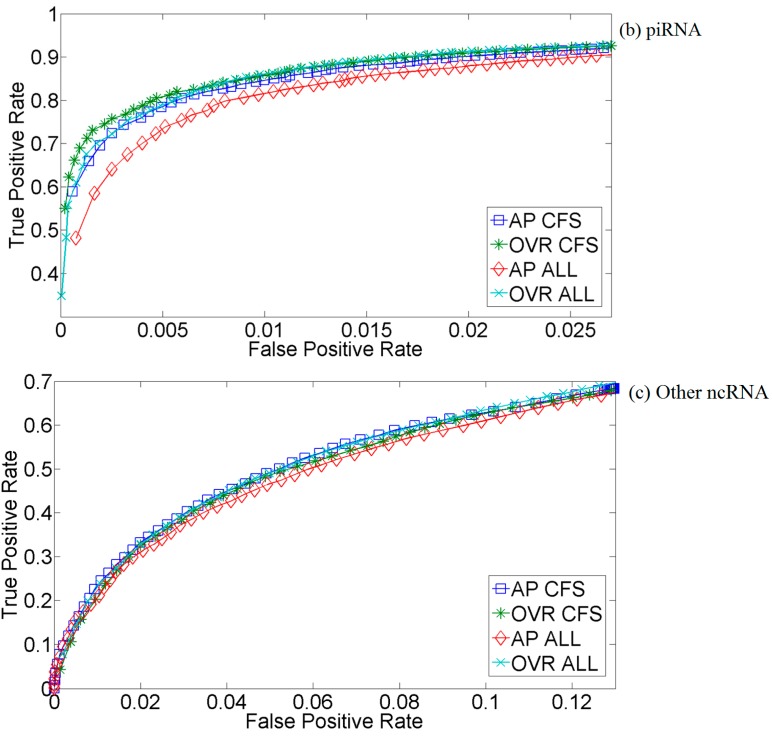
Three-fold cross-validation receiver operating characteristic (ROC) curve for correlation-based feature selection (CFS)-selected and all features (ALL) multiclass relevance units machine (McRUM) using the *L*_1_ Gaussian kernel with all-pairs (AP) or one *vs*. rest (OVR) decompositions. The ROC curves are generated from the observed false positive rate (FPR) and true positive rate (TPR) under varying posterior probability thresholds from 0.30 to 0.99 in increments of 0.01. (**a**) Results for microRNAs; (**b**) Results for piwi-interacting RNAs; (**c**) Results for other types of small RNAs.

Figure 1 illustrates the predictive performance of McRUMs with the *L*_1_ Gaussian kernel using all features or CFS-selected features. Two popular decompositions of a multiclass problem to a set of binary class problems are considered: all-pairs (AP) and one *vs*. rest (OVR). In the AP decomposition, a binary classifier is created for every pair of classes: (1) miRNA *vs.* piRNA; (2) miRNA *vs.* other ncRNA; and (3) piRNA *vs.* other ncRNA. On the other hand, the OVR decomposition creates a binary classifier for each class against all others: (1) miRNA *vs.* piRNA and other ncRNA; (2) piRNA *vs.* miRNA and other ncRNA; and (3) other ncRNA *vs.* miRNA and piRNA. The performance is measured using three-fold cross-validation. Figure 1b shows some advantages of using the OVR decomposition over AP. However, there is little difference between the models that use all features and CFS-selected features. This is surprising, as the order of the magnitude reduction of features and, thus, the dimensionality were expected to improve predictive performance, due to the relative contrast theory presented in [19] and since CFS should not be eliminating vital features. Nevertheless, the CFS analysis did provide insight into which features are actually necessary for prediction.

### 2.2. Kernel Selection

**Figure 2:**
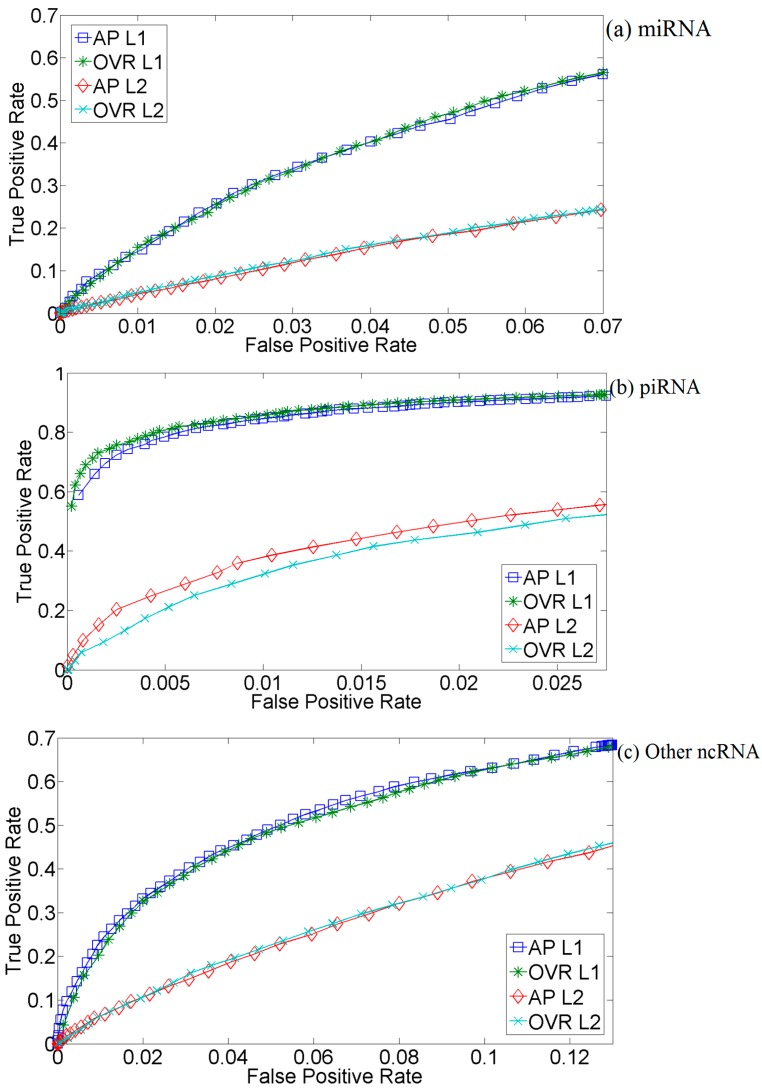
Three-fold cross-validation ROC curves for CFS McRUMs using *L*_1_ and *L*_2_ Gaussian kernels with all-pairs (AP) or one *vs*. rest (OVR) decompositions. The ROC curves are generated from observed FPR and TPR under varying posterior probability thresholds from 0.30 to 0.99 in increments of 0.01. (**a**) Results for microRNAs; (**b**) Results for piwi-interacting RNAs; (**c**) Results for other types of small RNAs.

We compare the use of *L*_1_ and the standard *L*_2_ Gaussian kernels with three-fold cross-validation using CFS-selected features. Figure 2 shows the significant increase of performance from using *L*_1_ rather than the standard *L*_2_ Gaussian kernel. For example, there is an increase to about a 0.9 true positive rate (TPR) using *L*_1_ from about 0.4 using *L*_2_ at a 0.01 false positive rate (FPR) for piRNA prediction (Figure 2b). Aggarwal *et al.* [19] studied the behavior of distance metrics in high dimensional clustering that explains the results we observe. They define a concept called the relative contrast, which measures how well you can discriminate between the nearest and furthest neighbor when using a particular distance metric. If relative contrast is too low, all points appear to be equally close by, which would be disastrous in classification, where the locality of data points is crucial. They go on to show that relative contrast using the *L*_1_ distance decays at a slower rate than the *L*_2_ distance as the dimension increases, and therefore, the *L*_1_ distance remains a viable metric at larger dimensions than the *L*_2_ distance. These theoretical results agree with our observations that the standard *L*_2_ Gaussian kernel evaluations in our problem are all near one, indicating low relative contrast, as all points in the dataset appear to be equally distant from (or similar to) each other. The increased relative contrast offered by using the *L*_1_ distance allows McRUM to more easily distinguish the data points belonging to different classes and, thus, improves predictive performance.

### 2.3. Comparison with SVM and piRNApredictor Using the Hold-Out Test Set

We use a hold-out test set to compare the predictive performance of McRUMs to the existing piRNApredictor software [15] and a Support Vector Machine (SVM) solution using the *L*_1_ Gaussian kernel and the CFS-selected features. We implement the *L*_1_ Gaussian kernel in the machine learning library, Weka [20], and use Weka’s implementation of sequential minimal optimization to train a SVM with logistic models. Weka’s implementation of multiclass SVM uses the AP decomposition to binary classification problems.

**Figure 3:**
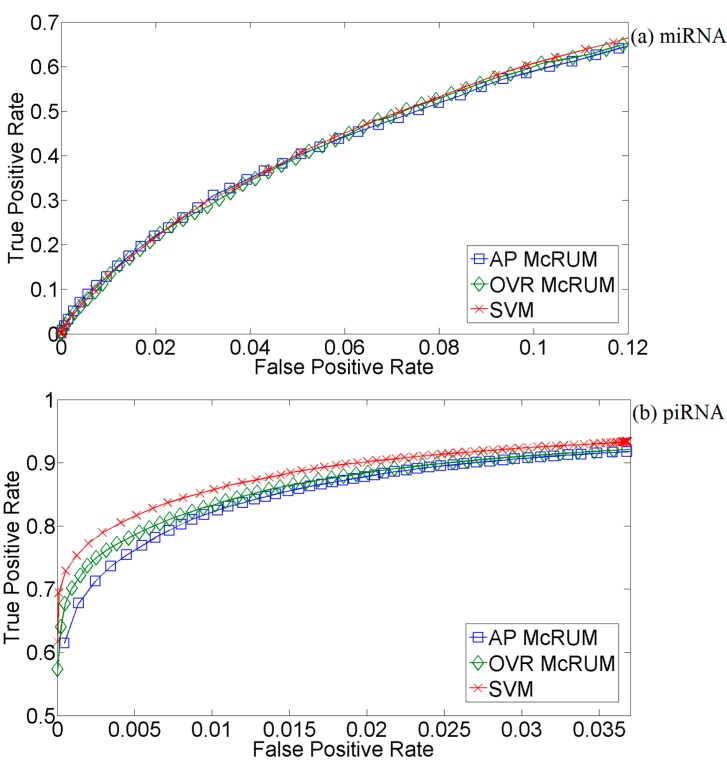

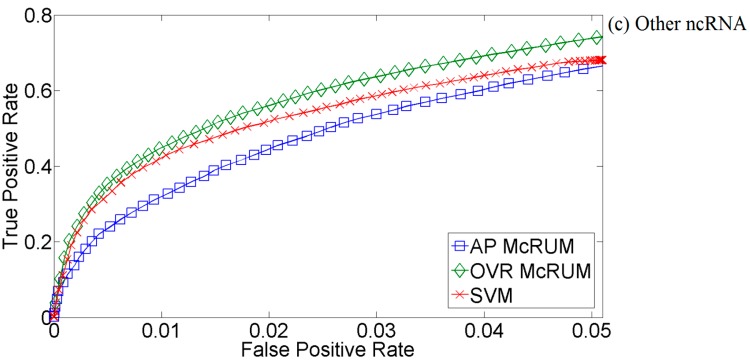
Test set ROC curves for McRUMs and SVM using the *L*_1_ Gaussian kernel. McRUMs use all-pairs (AP) or one *vs*. rest (OVR) decompositions. The ROC curves are generated from observed FPR and TPR under varying posterior probability thresholds from 0.30 to 0.99 in increments of 0.01; (**a**) Results for microRNAs; (**b**) Results for piwi-interacting RNAs; (**c**) Results for other types of small RNAs.

**Figure 4:**
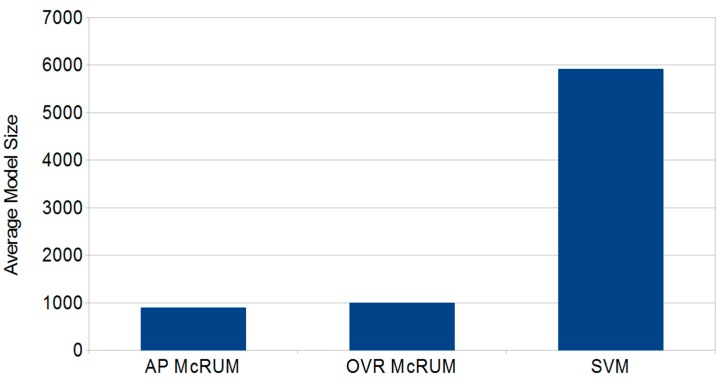
The average size (number of relevance units or support vectors) of the binary classification models for all-pairs (AP) and one *vs*. rest (OVR) McRUMs and SVM.

The predictive performance results of McRUMs and SVM are given in the ROC curves in Figure 3, which shows no clear superior method. Performance is nearly identical for miRNAs (Figure 3a). The SVM has a small advantage for piRNAs (Figure 3b), and the OVR McRUM has the advantage for other ncRNAs. The primary advantage of using McRUM models over the SVM model is the smaller models generated by McRUM, as illustrated in Figure 4. The SVM and both McRUM models are composed of three binary classification models. On average, a binary classification model of the SVM uses about six-times more terms (support vectors) in its linear model than McRUMs. Therefore, McRUM models are able to produce predictions at six-times the rate of the SVM, leading to faster RNA-seq analysis run-times.

**Figure 5:**
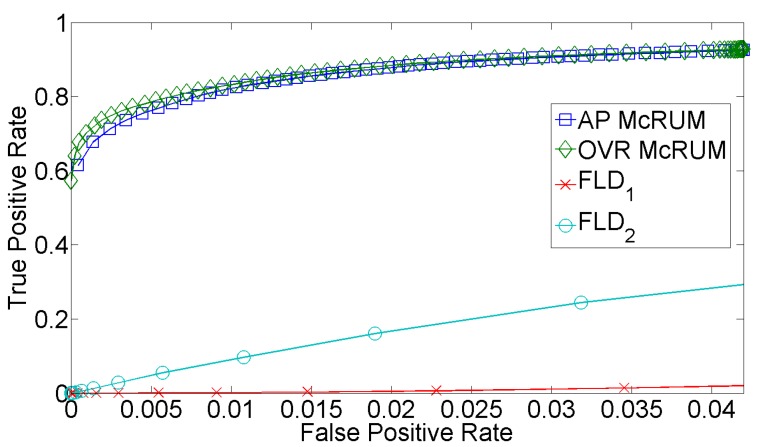
Test set ROC curves for all-pairs (AP) and one *vs*. rest (OVR) McRUMs and piRNApredictors: the original piRNApredictor (FLD_1_) (Fisher’s linear discriminant (FLD)) and a retrained piRNApredictor (FLD_2_). The ROC curves are generated from observed FPR and TPR under varying posterior probability thresholds from 0.30 to 0.99 in increments of 0.01.

The predictive performance of McRUMs for the piRNA case compared to the software piRNApredictor [15] is given in Figure 5. PiRNApredictor uses Fisher’s linear discriminant (FLD) classifier and makes predictions based on *k*-mer composition. Two variations of piRNApredictor are examined: the original provided by the authors and one built on our training dataset. Both McRUMs significantly outperform the piRNApredictors. One reason for the low performance of piRNApredictor is its use of an FLD’s linear model of the decision boundary between piRNA and other ncRNAs. We also observed poor performance using a linear SVM in our preliminary analysis [16]. Also noted in our preliminary analysis was the small representation of ncRNA sequences under 25 nt long in piRNApredictor’s training dataset. Only 4.67% of piRNApredictor’s training dataset were ncRNAs under 25 nt long. This weak representation of short ncRNAs may be the reason why the piRNApredictor trained on our dataset outperforms the original.

### 2.4. Comparison with miRPlex Using Experimental Datasets

We compare miRPlex [14] and our McRUMs using experimental RNA-seq datasets, since miRPlex requires information on sequence abundance and pairing sequences for the miRNA duplex prediction. This additional information is not provided in our training or hold-out test sets. Therefore, we consider the following four datasets: *Caenorhabditis elegans* (GSM297747), *Drosophila melanogaster* (GSM609220) and the gregarious (GSM317268) and solitary (GSM317269) phases of *Locusta migratoria*. While the ground truth is not known for experimental datasets, we are able to gain some insight into how miRPlex and McRUM perform individually and in conjunction with each other.

The primary disadvantage of miRPlex is its sheer computational complexity, as it requires invoking an RNA folding routine for every pair of sequences in the RNA-seq dataset, which may contain millions of sequences. To make miRPlex practical, the dataset must be filtered down to a smaller subset in some way. MiRPlex does this by allowing the user to specify the minimum abundance of the sequences to be considered. This removes lowly expressed sequences from the dataset, which can be an issue should the miRNA of interest occur at low expression levels. We also consider the use of McRUM as a filter for miRPlex, as an alternative to abundance filtering.

**Figure 6:**
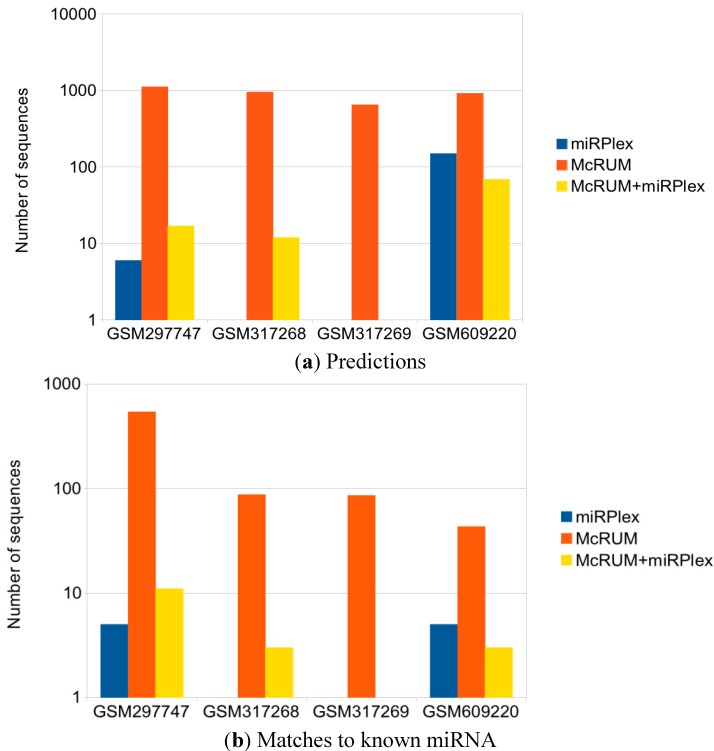
(**a**) Number of miRNA predictions for McRUM and miRPlex; (**b**) Number of miRNA predictions matching a known mature miRNA.

Due to computational constraints, we use a minimum abundance of 25 reads per million (RPM) for the GSM297747 and GSM609220 datasets and 40 RPM for GSM317268 and GSM317269 that have a larger number of unique sequences with high expression. For the McRUM results, we use a minimum posterior probability of 0.9 using an OVR-based McRUM model and a minimum abundance of two RPM to reduce noise. We also use the resulting McRUM outputs as the input to miRPlex with no abundance filter. This will allow miRPlex to pick out the McRUM results that have reasonable duplex pairings. The results are summarized in Figure 6a.

Due to the high number of unique sequences in the locust datasets, we were forced to use a high minimum abundance of 40 RPM. This is too stringent for miRPlex, and it is unable to detect any miRNAs. Using McRUM as the filter instead results in the detection of more miRNAs for two of the four datasets. MiRPlex still detects no miRNAs for GSM317269, while it detects less than half the amount of the abundance-filtered miRPlex for GSM609220. On the other hand, McRUM by itself detects about 1–2 orders of magnitude more miRNAs than the standard miRPlex.

To get a sense of the performance of these predictions, we look at how many of these predicted miRNAs closely match known miRNAs using a BLAST search against miRBase’s collection of mature miRNAs. We use NCBI’s blastn-short, which is optimized for sequences less of than 50 nucleotides [21]. A challenge with using BLAST on short sequences is that perfect or near perfect matches result in large *E*-values relative to analyses with long sequences. A perfect match of a length of 22 nt, roughly the length of a mature miRNA, only gets an *E*-value of 2 × 10^−7^. While only having a single mismatch, the *E*-value jumps up to 5 × 10^−5^, and two mismatches give an *E*-value of 2.8. This is troublesome because of the existence of isomiRs, which are variants of the canonical sequences given in miRBase. These isomiR variants include nucleotide substitutions, additions and trimming of the 5' and/or 3' end of the miRNA. We choose an *E*-value threshold of 5 × 10^−5^ to allow for a single mismatch in a 22-nt alignment in order to get some sensitivity to isomiRs, while still not preventing too many coincidental matches. While this analysis still remains rough and does not account for novel miRNAs, it does give a sense of how many of these predictions are reasonably close to known miRNAs. The results are summarized in Figure 6b and illustrate similar trends to that of Figure 6a. This shows that McRUM by itself can detect known miRNAs better than miRPlex using abundance or McRUM filtering by about an order of magnitude or more. This possibly comes with a number of false positives, but these can be reduced using a higher minimum posterior probability or by usage of miRPlex on McRUM’s predictions.

One additional potential reason for miRPlex’s under-performance is the aforementioned existence of isomiRs. miRPlex cannot account for the miRNAs being further modified after splitting apart from the duplex phase. The isomiR variants of the miRNAs may no longer be able to form a thermodynamically favorable duplex, and thus, miRPlex would not be able to detect these miRNAs. This is less of an issue with McRUM models, since the models do not consider the duplex and instead rely upon other miRNA features, such as nucleotide biases.

## 3. Experimental Section

### 3.1. Classification Methods

We use and compare the following two kernel-based classification methods: multiclass relevance units machine (McRUM) [16] and the support vector machine (SVM) [22] using the Gaussian kernel. The SVM is a popular method due to its high accuracy via a margin-maximizing framework. Probabilistic outputs will be generated from the SVM models by training a logistic regression model on the trained SVM’s outputs [23], as implemented in Weka [20]. Cross-validation is used to select the SVM parameters, the soft margin parameter *C* and the Gaussian kernel width.

McRUM is a new method that provides probabilistic outputs, high accuracy through its empirical Bayes foundations that mitigate overfitting and more parsimonious models than the SVM. McRUM decomposes an *n*-class classification problem into a set of binary classification problems that can be solved independently. In this study, we consider the popular and effective all-pairs (AP) and one *vs*. rest (OVR) decompositions. A binary classifier is generated for every pair of classes in the AP decomposition, resulting in *n*(*n* − 1)/2 binary classifiers. On the other hand, for each class in the OVR decomposition, the selected class is compared to the aggregation of all remaining classes. Therefore, the OVR decomposition consists of *n* binary classifiers.

Each binary classification problem is solved using the classification relevance units machine (CRUM) [24]. CRUM is a kernel-based classification method like SVM, which additionally provides probabilistic outputs that measure the uncertainty of the prediction. Unlike SVM, the number of kernel functions is specified, and overfitting is mitigated using empirical Bayes procedures. The centers of the Gaussian kernel functions are set to be the cluster centers determined by a k-means cluster analysis in this case. For this study, the same number of clusters/kernel functions is used for all CRUMs and is chosen using cross-validation. A logistic regression, like the Newton–Raphson algorithm with empirical Bayesian-based regularization, determines the weight of each Gaussian kernel. Finally, a linear-time algorithm aggregates the set of binary predictions from the trained CRUM models to produce the prediction for the original *n*-class problem using a normalized product of the binary outputs dictated by the decomposition.

### 3.2. Gaussian Kernels

In our previous analysis [16], we used the standard Gaussian radial basis function kernel for both McRUM and SVM,

(1) $$K\left(x,x^{\prime }\right)=\exp\left(\text{γ}{\left\|x-x^{\prime }\right\|}_{2}^{2}\right)$$

where γ controls the growth of the kernel, and the squared Euclidean distance of the two points **x** and **x**′ is considered. The use of Euclidean (*L*_2_) distance as its metric is not necessarily the optimal choice depending on the characteristics of the dataset, such as its dimensionality and sparseness. This is very well known in the clustering literature [19], but these results are not prevalently used in the classification literature. This is perhaps due to the technical difficulty that using a different metric in the Gaussian kernel may lead to an invalid kernel function and the fact that the popular SVM packages, LIBSVM (a machine learning library for SVM) [25], SVM*^light^* [26] and Weka [20], do not provide the built-in option to use different metrics in their Gaussian kernel implementations. However, it is known that the Gaussian kernel using the Manhattan (*L*_1_) distance leads to a valid kernel [27]. We investigate the use of the *L*_1_ distance-based Gaussian kernel over the standard *L*_2_ distance-based version with the *L*_1_ Gaussian kernel in a *d*-dimensional real vector space, defined as,

(2) $$K\left(x,x^{\prime }\right)=\exp\left(\text{γ}{\left\|x-x^{\prime }\right\|}_{1}\right)$$

where,

(3) $${\left\|x-x^{\prime }\right\|}_{1}=\sum_{i=1}^{d}\left|x_{i}-{x^{\prime }}_{i}\right|$$

### 3.3. Datasets

The small ncRNA dataset is gathered from miRBase’s collection of miRNA [28] and the NONCODE collection of piRNA [29], where both databases contain RNAs from a large variety of species. To represent other small RNAs that may be present in small RNA sequencing projects, we use the *Mus musculus* GSM314553 small RNA dataset from NCBI GEO database [30]. The mouse dataset is from a Dicer knockout mouse, which should not contain any mature miRNA due to Dicer’s important role in miRNA biogenesis. Any known mature miRNA and piRNA in this dataset will be filtered out. This should provide a more realistic representation of other small RNAs than our previous effort [16] that used random short fragments of other ncRNAs from the NONCODE database. Each of these three sets is individually reduced using the sequence clustering package CD-HIT [31] to remove sequences with 80% or higher identity. This helps reduce the evolutionary correlations among the data and improves the generalization of McRUM and SVM models, which assumes an independent sample.

After redundancy reduction, the dataset consists of 13,891 miRNA, 81,147 piRNA and 190,761 other ncRNA sequences. We randomly set aside 20% of the miRNA sequences for the hold-out test set and the remaining 80% for the training set. We randomly select an equal number of piRNA and other ncRNA sequences for training to keep the training sets balanced. All remaining piRNA and other ncRNA sequences are used in the hold-out test set.

For comparison with miRPlex, four public experimental RNA-seq datasets are used. These datasets have already been preprocessed to remove some of the noise. Table 1 summarizes information about the datasets. In the datasets, sequences are filtered based on minimum RPM due to computational constraints. As described in Section 2.4, we use a minimum of 25 RPM for GSM297747 and GSM609220 and 40 RPM for GSM317268 and GSM317269. This further reduces the presence of noisy sequences and, thus, potential false predictions.

**Table 1:** Summary of experimental datasets.

Dataset	Species	Library Size	# of Unique Sequences	# of Filtered Sequences
GSM297747	*Caenorhabditis elegans*	5 million	371,937	1353
GSM317268	*Locusta migratoria*	1 million	228,526	2091
GSM317269	*Locusta migratoria*	2 million	429,041	1668
GSM609220	*Drosophila melanogaster*	14 million	78,236	1349

### 3.4. Features

Since mature miRNAs and piRNAs lack strong secondary structures, we will represent each ncRNA using *k*-mers, for *k* = 1 through 5. For each value of *k*, the number of occurrences of each type of *k*-mer is computed and normalized. If we view each ncRNA sequence as a text document, this *k*-mer approach is analogous to the “bag of words” approach in document classification. Our previous analysis [16] suggests that this *k*-mer approach suffices for identifying piRNAs, but is poor at identifying miRNAs.

To improve the performance with regards to miRNAs, we consider additional features. It is known that miRNAs have a strong bias toward the U nucleotide in the first position of their sequences [14]. We therefore propose to use four binary features for the four possible RNA nucleotides, A, C, G and U, to mark the identity of the first nucleotide of the read. It is also known that the target mRNAs of a miRNA need to be physically accessible, particularly to the first eight positions of the miRNA, which is called the seed region [32]. Accessibility is correlated with the nucleotide composition of the mRNA target region and, thus, the miRNA’s nucleotide composition. The composition of the entire miRNA is already captured by the *k*-mer features, but to emphasize the importance of the seed region, we propose the addition of separate *k*-mer features computed over the seed region only. Lastly, we also consider the AU score proposed in [18] as another feature related to accessibility. The AU score is a measure of AU content weighed by the distance of the A/U occurrence to the seed region.

Using *k* = 1 and 2 only for the seed region *k*-mer composition features, we have a grand total of 1389 features. Since this is high dimensional, we may be able to achieve improved results through feature selection. We use CFS [17] to select a subset of features on which to build classifier models. The core idea of CFS is that the best subset of features is the set of those that are highly correlated with the class and minimally correlated with each other. In other words, CFS seeks to find features that are maximally relevant and minimally redundant. We chose CFS because it is applicable to non-linear models through its use of mutual information, a non-linear measure of correlation. To prevent overfitting through feature selection, we use CFS in the cross-validation context.

## 4. Conclusions

We proposed an approach to the prediction of mature miRNA and piRNA that relies primarily on the nucleotide composition of the read. This approach avoids the need of direct genome reference and genomic information of other species closely related to the target. Furthermore, it does not require computationally expensive information or features, such as pairwise folding of the reads required by existing methods. A correlation-based feature selection scheme is employed and achieves about an order of magnitude reduction in the dimensionality of feature space, which not only makes the training of prediction models efficient, but also provides insight into the set of features important for the prediction. Using the McRUM classification method, compact models suitable for large-scale analyses are built using databases of known mature miRNA and piRNA. Cross-validation results show that the use of an *L*_1_-based Gaussian kernel can double the true positive rate compared to the standard *L*_2_-based Gaussian kernel. Analysis of the hold-out test set shows that our approach can increase the true positive rate for piRNA by at most 60% compared to piRNApredictor. Finally, we show that our approach can detect about an order of magnitude or more of the known miRNAs than miRPlex using experimental datasets.

In the future, we plan to study the possibility of applying the proposed approach to detecting miRNA-targeted genes and predicting the miRNA binding sites along the target mRNA. Since the number of experimentally verified miRNA-mRNA pairs has been increasing, data-driven approaches to address these problems become viable and have drawn much interest. Applying our method to the target gene and site predictions will also include studies on the identification and selection of efficient features for these tasks.
